# The influence of trigger factors on hereditary angioedema due to C1-inhibitor deficiency

**DOI:** 10.1186/1750-1172-9-44

**Published:** 2014-03-28

**Authors:** Zsuzsanna Zotter, Dorottya Csuka, Erika Szabó, Ibolya Czaller, Zsuzsanna Nébenführer, György Temesszentandrási, George Fust, Lilian Varga, Henriette Farkas

**Affiliations:** 1 3rd Department of Internal Medicine, Semmelweis University, Budapest, Hungary

**Keywords:** Hereditary angioedema, C1-Inhibitor deficiency, Trigger factor, Subcutaneous/submucosal attack

## Abstract

**Background:**

Hereditary angioedema (HAE) resulting from C1-inhibitor deficiency is characterized by attacks of subcutaneous and submucosal edema. Many factors have been presumed to induce edema. Our study analyzed these factors in a fairly large patient population.

**Methods:**

In the first stage of our study, we analyzed the data recorded by 92 subjects in their patient diaries over seven years. The second phase included 27 HAE patients, who had been completing the diary entry ‘Trigger factors’ every day for seven months whether or not they had experienced an attack.

**Results:**

During the initial stage, 91% of the subjects described some factor possibly related to the onset of an attack. They could identify a trigger factor – most commonly (21%) mental stress – in 30% of the 3176 attacks. We found a significant (p < 0.001) difference in the distribution of the trigger factors of the edematous attacks of different locations. The 27 participants of the second phase identified 882 potential trigger factors and recorded 365 attacks. Of these, 246 (67%) occurred on days when the patients identified a potential trigger factor. The likelihood of edema-formation associated with the latter was as follows: menstruation – 63%, infection – 38%, mental stress – 26%, physical exertion – 25%, meteorological changes – 21%, fatigue – 17%.

**Conclusion:**

This analysis of the trigger factors explored, for the first time, their potential role in inducing HAE attacks. Our findings might open new perspectives in extending the indications for edema-prophylaxis, and could contribute to a better understanding of the pathomechanism of HAE attacks.

## Introduction

Hereditary angioedema (HAE) due to C1-INH deficiency (HAE-C1-INH) is a rare autosomal dominant disorder. The deficiency of C1-INH may cause activation of four closely interrelated (complement, contact-kinin, coagulation, and fibrinolytic) enzyme cascade systems. This may lead to the release of bradykinin, resulting in recurrent, paroxysmal angioedema in the subcutis and/or in the submucosa of the gastrointestinal tract and of the upper airways. [1-4]. Acute upper airway obstruction caused by edema may lead to suffocation [5]. The management of the disease consists of the prevention and therapy of edematous attacks. The diagnosis, management, and follow-up of HAE-C1-INH patients are guided by international recommendations and consensus guidelines based on the latest scientific findings [6-8].

The genetics of HAE-C1-INH are complex; around 300 mutations have been described so far (see http://hae.enzim.hu). The correlation between the clinical phenotype and the genetic background is poor [9-11].

In a proportion of episodes, there are certain background events, which the patients regard as triggers of attacks. Elucidating the role of these “trigger” factors in initiating edema formation can help to understand why these attacks occur. According to empirical observations, physical exertion, mechanical trauma, mental stress, infection, menstruation, pregnancy, medical interventions, changes of weather, and certain medicinal products [3,12-16] are associated with an increased risk of a HAE attack. In view of the pathomechanism of edema formation, the individual trigger factors might influence this process at its different stages. The role of trigger factors in the pathophysiology of edema has not yet been clarified.

Although many of these factors are ubiquitous in everyday life, HAE-C1-INH patients do not experience an attack every day. The objective of our study was to appraise the incidence of various trigger factors, along with their effect on edematous attacks.

## Methods

### Stage I of the study

During the initial phase of the study from 2004 to 2010, we performed diagnostic evaluation (family history, symptoms, genetic testing, and complement studies) of 140 HAE-C1-INH patients at the Hungarian HAE Center. Our survey reviewed the entries made by the subjects in their standardized Patient Diaries over 7 years. At the initial visit, patients received individualized information on the nature of the disease and its possible manifestations. The locations, severity, trigger factors, and treatment of HAE attacks were also discussed. Every year, each patient received a standardized Patient Diary. The latter was intended for recording the date of onset, severity, location, and potential trigger factors of the attacks according to standard criteria, along with the treatment received. The Patient Diaries were reviewed at the annual follow-up visits (including evaluation of the relevance and regularity the entries). At the end of each year, the patients returned their diaries by mail or in person. Overall, we cannot tell how accurate these records are as far as the properties of the events are concerned. The patients affirmed that they had made efforts to record every attack in the diary. Nevertheless, ascertaining the occurrence of an attack was feasible only in the case of those that required treatment with C1-INH concentrate. This medicinal product was administered by medical professionals who documented their cases and furthermore, the usage of this product is monitored by the National Health Insurance Funds Administration. We found an 88-per-cent agreement between the entries of the patient diaries and of the medical records, regarding treatment with C1-INH concentrate.

Analyzing these data, we appraised the characteristics (e.g. location, trigger factors) of the attacks, as well as their seasonal and monthly distribution.

### Stage II of the study

In the second, prospective stage, we studied 27 patients. These subjects recorded the occurrence of possible trigger factors every day for seven months –whether or not they had experienced an attack. In particular, these patients made a note every day of the occurrence of events empirically associated with an increased risk of edema formation. The subjects had received a list of possible trigger factors along with the patient diary, which they kept from July until December 2011. Thus, patient data from 7 months were analyzed. In the second phase of the study, patients were contacted monthly by telephone to support their compliance with the instructions on keeping the Patient Diary.

### Statistical methods

Statistical analyses were performed with the GraphPad Prism 4.0 program (GraphPad Software Inc, San Diego, CA, http://www.graphpad.com). We used the Chi-square test to analyze differences between the distribution of trigger factors and of edema locations. To explore annual distribution, we summarized the number of attacks occurring during the four temperate-climate seasons. Furthermore, we summarized the monthly numbers of the attacks recorded each year, from 2004 to 2010. As changes in patient compliance may introduce differences, we calculated weighted averages. From these, we determined the mean annual number of attacks and then, identified the months with a higher weighted average. Next, we identified the months with a higher than average number of attacks during the seven-year long phase I of the study. All hypotheses were tested against two-directional alternatives and p < 0.05 was considered significant.

## Results

### Stage I of the study

Of the 140 HAE-C1-INH patients, six remained symptom-free until the end of the study. Of the 134 subjects with symptoms, 92 (56 females and 36 males, mean age 38.35 [range: 12 to 48] years) made records suitable for analysis to identify trigger factors.

During the seven-year long observation period, these 92 patients recorded information on 3176 episodes. Sixty-five per cent (2187/3176) of these were subcutaneous, and 35% (1151/3176) were submucosal (937 abdominal and 214 upper airway) attacks. Seventy-one per cent (2256/3176) of the attacks occurred in female patients. In 162 instances, edema formation affected multiple locations – that is, involved subcutaneous and submucosal tissues concomitantly. Eight of the 92 patients could not detect any potential trigger in their lives, whereas 84 suspected at least one such factor.

#### ***The trigger factors identified by the patients***

The subjects identified several trigger factors (Table 1), most commonly physical exertion (66 subjects), followed by mental stress, and mechanical trauma (55–55 patients).

**Table 1:** The trigger factors of HAE attacks, ranked by frequency

**Trigger factor**	**Number of involved patients (Σn = 92)**
Physical exertion	66
Mental stress	55
Mechanical trauma	55
Infection	41
Weather changes	29
Menstruation	25
Foodstuffs	18
Dental procedures	15
Fatigue/exhaustion	8
Medical procedures	7
Pregnancy	6
Estrogen-containing oral contraceptive use	3
**Others**	
Ovulation	2
Insect bites	2
Allergy	2
Cosmetics	2
Treatment with ACEI	1
Thermal injury	1
Gastric acid hypersecretion	1
Prolonged voluntary suppression of micturition	1

#### ***Trigger factors and total attack number***

The subjects could identify a trigger factor in 30% (953/3176) of all recorded attacks, and in 23.64% of subcutaneous, 38.13% of abdominal, and 28.50% of upper airway episodes. The following trigger factors were identified: mental stress (21%), physical exertion (17%), weather changes (15%), menstruation (18% in the female population), infection (11%), trauma (11%), fatigue (6%), pregnancy (4% in the female population), others (3%) (Table 2).

**Table 2:** The distribution of trigger factors by attack locations

	**Attacks with known trigger factor**	**Subcutaneous episodes**	**Abdominal episodes**	**Upper airway episodes**
Mental stress	21	15	27	22
Menstruation	18	11	17	26
Physical exertion	17	24	13	-
Weather changes	15	14	15	8
Infection	11	10	12	26
Mechanical trauma	11	17	1	7
Fatigue	6	4	8	8
Dental procedures	-	2	-	-
Foodstuffs	-	2	6	-
Other	3	1	1	3

The values presented in the table are percentages (%).

#### ***Trigger factors and location of attacks***

We analyzed the attacks of different (subcutaneous, abdominal, or upper airway) locations according to the distribution of their trigger factors (Table 2). Subcutaneous edema was most often induced by physical exertion (24%). The leading trigger factor of abdominal attacks was mental stress (27%), whereas in the case of upper airway edema, infection (26%) was followed by menstruation (26%). The chi-square test showed a significant (p < 0.0001) difference between the distributions of the trigger factors of the edematous attacks of different locations.

#### ***The monthly and seasonal distribution of attacks***

The weighted average of the number of submucosal attacks was higher in January, August, and October than in the other months of the year. The number of subcutaneous attacks peaked in November, and episodes without an identifiable trigger factor occurred most often in March and October. We observed a similar distribution of the attacks induced by mental stress.

### Stage II of the study

The second, prospective stage of our study included 27 patients (5 males and 22 females, mean age: 36.26 [range: 9 to 58] years). These subjects had been making diary entries to record possible trigger factors every day for seven months – whether or not they had experienced an attack. During the seven months of Stage II, the patients identified 882 possible trigger factors and recorded 365 attacks. Two hundred forty-six of the latter occurred on days when the patients could identify at least one “likely” trigger factor. Overall, 67% of the attacks were associated with a suspected trigger. Of the 365 attacks, 234 affected the subcutis, 114 were abdominal, and 17 occurred in the upper airways.

#### ***The likelihood of an attack associated with individual trigger factors***

We examined the association between the individual trigger factors and the occurrence of attacks. The following ratios were observed: menstruation 38/60 (63%), infection 28/74 (38%), mental stress 53/199 (26%), physical exertion 41/167 (25%), weather changes 58/274 (21%), and fatigue 16/92 (17%). According to the patients, trauma occurred on seven occasions, and an attack ensued in five of these. Food was the suspect trigger factor in nine cases, of which seven were associated with an attack, which indeed might have resulted from the consumption of the incriminated foodstuff. The majority of the infections involved the upper airways. One patient reported urinary tract infection, whereas enteritis was the likely trigger factor in another. Table 3 shows the number of attacks induced by the individual triggering factors for each patient.

**Table 3:** The number of attacks induced by the individual triggering factors for each patient

**Patient no.**	**Menstruation**	**Infection**	**Mental stress**	**Physical exertion**	**Weather changes**	**Fatigue**	**Unknown**
1	8/8	1/2	3/6	1/2	2/16	1/2	4
2	-	1/1	0/6	-	0/7	-	1
3	3/5	0/8	2/7	1/3	4/48	1/3	1
4	7/7	-	-	-	-	-	42
5	0/1	1/15	3/14	-	-	2/21	-
6	-	5/8	6/9	2/4	10/30	1/4	2
7	0/7	0/1	1/1	1/24	-	0/1	-
8	-	1/1	5/9	1/3	5/14	2/6	15
9	-	-	-	4/4	-	-	-
10	4/4	-	7/7	-	-	-	14
11	3/4	0/4	3/48	5/53	2/3	0/2	-
12	-	3/5	1/2	6/27	15/65	2/13	-
13	-	5/8	7/22	-	0/2	0/1	-
14	2/4	0/1	-	-	1/1	-	-
15	-	-	4/14	2/2	1/1	0/1	4
16 M	-	-	-	5/5	-	-	1
17 M	-	4/4	-	0/2	-	-	3
18	-	1/2	1/7	0/6	0/3	0/12	2
19	0/1	0/2	0/17	0/4	0/11	0/11	-
20	3/6	1/3	3/15	0/2	0/6	3/7	3
21	-	0/3	1/4	-	-	0/4	-
22 M	-	-	-	8/8	9/9	-	4
23 M	-	2/2	-	1/1	3/3	1/1	12
24	5/5	-	2/2	-	3/3	-	6
25	2/2	1/1	-	3/12	-	-	4
26	1/6	0/1	0/2	-	1/14	-	-
27 M	-	2/3	4/7	1/5	2/38	3/3	1

The letter ‘M’ indicates male patients.

#### ***The relationship between trigger factors and attack locations***

We examined the individual trigger factors of attacks involving different locations. Table 4 shows the trigger factors according to the location of the attack. Physical exertion usually induced a subcutaneous attack, whereas menstruation triggered submucosal edema formation. The chi-square test showed a significant (p < 0.0001) relationship between the type of the trigger factor and the location of the attack.

**Table 4:** The distribution of the attacks induced by various trigger factors, by location

**Trigger factor**	**Subcutaneous episodes**	**Abdominal episodes**	**Upper airway episodes**
Menstruation	11	21	6
Mental stress	35	12	6
Physical exertion	38	3	0
Weather changes	49	8	1
Infection	14	10	4
Mechanical trauma	5	0	0
Fatigue	15	1	0
Foodstuffs	0	7	0
Non-explorable trigger factor	67	52	0

The values presented in the table are percentages (%).

#### ***The likelihood of edematous attacks in patients undergoing/not receiving long-term prophylaxis***

The likelihood of an attack triggered by infection, stress, physical exertion, and weather changes was higher in patients undergoing than in those not receiving long-term prophylaxis (LTP) Table 5. Such a comparison was impossible during Stage I of the study, because the changes made to the therapeutic regimen during the observation period precluded defining sufficiently homogenous treatment groups.

**Table 5:** The likelihood of edematous attacks in patients undergoing/not receiving long-term prophylaxis

**Trigger factor**	**Patients not receiving long-term prophylaxis**	**Patients receiving long-term prophylaxis**	**Whole population**
Menstruation	62.5%	65%	63%
Infection	23.7%	52.8%	38%
Mental stress	12.9%	53.7%	26%
Physical exertion	12%	54%	25%
Weather changes	15.4%	28%	21%
Fatigue	18%	16.1%	17%
Non-explorable trigger factor	35.3%	32.5%	32.6%

## Discussion

Our study explored trigger factors during the long-term follow-up of our patient population. This is the first, systematic review of the possible role of these factors in HAE attacks. Ninety-one per cent of the subjects could identify a trigger factor – most often physical exertion (71%), mental stress (59%), and mechanical trauma (59%). The patients detected a trigger factor – most commonly mental stress – in one-third of the edematous episodes they had experienced. Therefore, providing mental health support or antidepressant treatment may be considered when necessary. It is a novel observation that in the case of attacks with a particular trigger, the patients held weather changes responsible in one-sixth of all these episodes. A variety of major trigger factors induced attacks in different locations.

The subjects suspected a trigger factor most often in relation to abdominal attacks. Physical exertion most commonly provoked subcutaneous edema, whereas mental stress induced abdominal attacks for the most part. Infection and menstruation were responsible for the majority of upper airway episodes. The frequency of mucosal symptoms was lower than usually reported [17-19]. Episodes involving multiple locations were also less common. The background of these phenomena is not clear; possible explanations include ethnic diversity and/or differences in lifestyle. We detected a similar trend in a previous study conducted on a group of Hungarian and German patients: the incidence of abdominal attacks was significantly – three times – higher among German patients [20].

The presence of a trigger factor, suspected by the patient as the cause of edema, does not necessarily lead to an actual attack. Among the trigger factors identified, menstruation was most likely to lead to edema formation (in 68% of cases). In a subset of patients, menstruation was a typical inducer of edematous episodes. In this study, we found that certain trigger factors were more likely to induce attacks in patients undergoing than in those not receiving long-term prophylaxis. This is possibly explained by the fact that the former have more severe disease (which requires LTP) and as such, they are more susceptible to the influence of trigger factors. Counseling these patients about possible trigger factors is thus indispensable, as it enables them to prepare for future attacks.

The knowledge about the role of trigger factors makes it possible to change the lifestyle of the patient and to introduce individualized management.

### Conclusions

The exact role of trigger factors needs further clarification, because they do not necessarily induce an attack. Thus, predicting the occurrence of edematous episodes in HAE-C1-INH patients remains unfeasible. Nevertheless, with the knowledge of these factors is important, because primary prevention – that is, avoiding specific trigger factors – is possible in a proportion of cases. According to our findings, menstruation – a potent trigger factor – appears another suitable target for short-term drug prophylaxis. To determine the optimum timing of the latter, the propensity for edema formation during the menstrual cycle should be explored further. Early diagnosis, the identification of trigger factors, timely recognition of prodromal and initial symptoms, counseling, and state-of-the art management may help HAE-C1-INH patients to live a normal life.

## Abbreviations

HAE: Hereditary angioedema; C1-INH: C1-Inhibitor; HAE-C1-INH: Hereditary angioedema due to C1-inhibitor deficiency; FXII: Factor XII; ACEI: Angiotensin converting enzyme inhibitor.

## Competing interest

Henriette Farkas has received consultancy/speaker fees and honoraria from Shire Human Genetic Therapies Inc., Pharming, Viropharma, and CSL Behring. Dorottya Csuka has received travel grants from CSL Behring, Shire Human Genetic Therapies Inc, and Viropharma. Lilian Varga has received travel grants from CSL Behring, and Shire Human Genetic Therapies Inc.

## Authors’ contributions

ZZ: concept and design of the study; accumulation of data; analysis and interpretation of the data; and writing/critical revision of the manuscript. DC: concept and design of the study; accumulation of data (when applicable); analysis and interpretation of the data; and writing/critical revision of the manuscript. ES: accumulation of data; analysis and interpretation of the data; and writing/critical revision of the manuscript. IC: accumulation of data; analysis and interpretation of the data; and writing/critical revision of the manuscript.ZN: accumulation of data; analysis and interpretation of the data; and writing/critical revision of the manuscript. GT: accumulation of data; analysis and interpretation of the data; and writing/critical revision of the manuscript.GF: concept and design of the study; analysis and interpretation of the data; and writing/critical revision of the manuscript. LV: concept and design of the study; accumulation of data; analysis and interpretation of the data; and writing/critical revision of the manuscript. HF: concept and design of the study; accumulation of data; analysis and interpretation of the data; and writing/critical revision of the manuscript. All authors read and approved the final manuscript.
